# Uso do SAPS 3 no escore *NUTrition RIsk in the Critically ill* modificado tem precisão preditiva comparável ao uso do APACHE II como marcador de gravidade

**DOI:** 10.5935/0103-507X.20210064

**Published:** 2021

**Authors:** Valeska Fernandes Pasinato, Oellen Stuani Franzosi, Sérgio Henrique Loss, Diego Silva Leite Nunes, Kelly Carraro Foletto, Gabriela Soranço Salazar, Silvia Regina Rios Vieira

**Affiliations:** 1 Programa de Pós-Graduação em Ciências Médicas, Faculdade de Medicina, Universidade Federal do Rio Grande do Sul - Porto Alegre (RS), Brasil.; 2 Divisão de Nutrição e Dietética, Hospital de Clínicas de Porto Alegre, Universidade Federal do Rio Grande do Sul - Porto Alegre (RS), Brasil.; 3 Unidade de Terapia Intensiva, Hospital de Clínicas de Porto Alegre, Universidade Federal do Rio Grande do Sul - Porto Alegre (RS), Brasil.; 4 Departamento de Clínica Médica, Universidade Federal do Rio Grande do Sul - Porto Alegre (RS), Brasil.

**Keywords:** Avaliação nutricional, Cuidados críticos, APACHE, Escore fisiológico agudo simplificado, Mortalidade, Índice de gravidade de doença

## Abstract

**Objetivo:**

Avaliar o *Simplified Acute Physiology Score 3* (SAPS 3) como substituto do *Acute Physiology and Chronic Health Evaluation II* (APACHE II) como marcador de gravidade na versão modificada do escore *NUTrition RIsk in the Critically ill* (mNUTRIC; sem interleucina 6), com base em uma análise de sua capacidade discriminativa para predição de mortalidade hospitalar.

**Métodos:**

Este estudo de coorte retrospectiva avaliou 1.516 pacientes adultos internados em uma unidade de terapia intensiva de um hospital geral privado entre abril de 2017 e janeiro de 2018. A avaliação de desempenho incluiu as análises Kappa de Fleiss e correlação de Pearson. A capacidade discriminativa para estimar a mortalidade hospitalar foi avaliada com a curva Característica de Operação do Receptor.

**Resultados:**

A amostra foi dividida aleatoriamente em dois terços para o desenvolvimento do modelo (n = 1.025; idade 72 [57 - 83]; 52,4% masculino) e um terço para avaliação do desempenho (n = 490; idade 72 [57 - 83]; 50,8 % masculino). A concordância com o mNUTRIC foi Kappa de 0,563 (p < 0,001), e a correlação entre os instrumentos foi correlação de Pearson de 0,804 (p < 0,001). A ferramenta mostrou bom desempenho para prever a mortalidade hospitalar (área sob a curva de 0,825 [0,787 - 0,863] p < 0,001).

**Conclusão:**

A substituição do APACHE II pelo SAPS 3 como marcador de gravidade no escore mNUTRIC mostrou bom desempenho para predizer a mortalidade hospitalar. Esses dados fornecem a primeira evidência sobre a validade da substituição do APACHE II pelo SAPS 3 no mNUTRIC como marcador de gravidade. São necessários estudos multicêntricos e análises adicionais dos parâmetros de adequação nutricional.

## INTRODUCTION

The NUTrition RIsk in the Critically ill (NUTRIC) scoring system is the only nutritional screening tool developed specifically for critically ill patients.^(1)^ It was proposed by Heyland et al. for assessing the risk of adverse events (i.e., mortality, days on mechanical ventilation - MV), which are potentially modifiable by adequate nutritional intervention.^(1)^ The tool is based on a conceptual model that addresses current lines of thought on malnutrition in adult patients and includes disease severity, chronic starvation, and inflammation, stressing their influence on the nutritional and prognostic status of a patient on intensive care unit (ICU) admission.^(1)^ The instrument has been modified and validated without interleukin-6, which was included in the first version but then removed due to measurement difficulties in most centers. When the interleukin 6 measurement was removed, Rahman et al. did not observe any clinically or statistically significant changes in their data, recommending the removal of the score marker without prejudice to the score.^(2)^ The NUTRIC scoring system is recommended by national and international guidelines^(3, 4)^ and identifies that approximately half of patients admitted to the ICU have high nutritional risk.^(5)^

The NUTRIC system uses the Acute Physiology and Chronic Health Evaluation (APACHE) II score as a marker of severity and prognosis. However, there is a new generation of prognostic scores that are widely available and can be applied earlier and more easily, such as the Simplified Acute Physiology Score (SAPS) 3.^(6^-^8)^ The SAPS 3 system was developed in a global cohort and consists of 20 variables divided into demographic data, physiologic parameters, and reasons for ICU admission. Total SAPS 3 score may range from 16 to 217 points.^(8)^ It has the advantage of calculating the probability of death within the first hour of ICU admission and calibrating it according to the world region. Because of these characteristics, it has been incorporated into several clinical research protocols in ICU settings.

With the increasing adherence to the SAPS 3 rather than APACHE II as a severity score in ICUs, the use of NUTRIC score modified version (mNUTRIC) as a nutritional screening tool in clinical settings is finding difficulties.^(8, 9)^ The unavailability of APACHE II data and the time required to calculate this score as a step prior to performing the mNUTRIC evaluation make the time required for its application long, an unwanted feature for nutritional screening tools. The SAPS 3 is a prognostic system and predicts mortality as the APACHE II score. For the mNUTRIC, we hypothesized that using SAPS 3 instead of APACHE II as a severity marker results in a comparable predictive accuracy of mortality. We aimed to contribute to the provision of the first evidence about the validity of the substitution of APACHE II by SAPS 3 in the mNUTRIC as a marker of severity.

## METHODS

This retrospective cohort study included patients admitted to an ICU of a private general hospital in Brazil who stayed more than 24 hours from April 2017 to January 2018. They underwent nutritional risk assessment on ICU admission using the mNUTRIC score in the first 24 - 48 hours.

The study was conducted in accordance with the Declaration of Helsinki and was approved by the local research ethics committee (protocol #18-0271). The authors signed an agreement to preserve patient and staff anonymity related to the use of these data. Given the characteristics of the study, patient consent was waived.

### Data collection

The following epidemiological and clinical variables were collected: age, sex, body mass index (BMI), Sequential Organ Failure Assessment (SOFA), APACHE II, SAPS 3, use of MV, place of origin (before ICU admission), reason for ICU admission, lengths of ICU and hospital stay, and ICU and in-hospital mortality.

Nutritional risk assessment was performed using the mNUTRIC score, whose final score consists of the sum of scores assigned to the following components: age, APACHE II, SOFA, number of comorbidities, and length of hospital stay before ICU admission. Classification was based on the system proposed for the modified version: a low score was zero to four points (low risk), and a high score was ≥ 5 to 9 points (high risk).^(2)^

### Substitution of APACHE II by SAPS 3 in mNUTRIC

Simplified Acute Physiology Score 3 scoring ranges were defined using APACHE II cutoff points from linear regression modeling and comparison in the Receiver Operating Characteristic (ROC) curve for in-hospital mortality. The score assigned to the ranges of the SAPS 3 component was maintained according to the original instrument (zero to three points). Patients were classified as high nutritional risk when the score was ≥ 5 - 9 points. To validate this model, all-cause in-hospital mortality was used as the outcome.

### Statistical analysis

The sample size was calculated based on the study of Silva Junior et al.,^(8)^ which evaluated whether SAPS 3 is applicable to Brazilian ICUs and found a 75.8% sensitivity in the discrimination between survivors and nonsurvivors. Considering a 0.7 sensitivity with a 0.1 precision and a 0.55 prevalence of mortality (obtained from institutional data), the minimum number of patients was 148.

Quantitative variables were summarized as medians and interquartile ranges. Qualitative variables were expressed as absolute and relative frequencies. The Shapiro-Wilk test was used to assess the normality of variables. Poisson regression was used to assess the relationship between severity scores and in-hospital mortality, adjusted for number of comorbidities, age, sex, place of admission, use of MV, and BMI. Correlations between instruments were analyzed using the Pearson correlation coefficient.

Agreement between the instruments on nutritional risk classification was assessed using Fleiss’ kappa (k). This index ranges from zero to one and considers < 0.2 low agreement, 0.2 to 0.4 fair agreement, 0.4 to 0.6 moderate agreement, 0.6 to 0.8 substantial agreement, and > 0.8 almost perfect agreement.

The ability to predict in-hospital mortality in a model composed of the SAPS 3 score was analyzed using the area under the ROC curve (AUC) and 95% confidence intervals (95%CI). The level of significance was set at 5%. The predictive validity of the proposed model versus the mNUTRIC score was assessed using Poisson regression with robust variance for in-hospital mortality, adjusting for age and sex. For data analysis, the Statistical Package for the Social Sciences (SPSS) software, version 21.0, was used.

## RESULTS

From April 2017 to January 2018, 1,516 patients were considered eligible. The sample was randomly divided into two-thirds for model development (n = 1,025) and one-third for model performance evaluation (n = 490). Patients’ characteristics are described in table 1.

A correlation was observed between APACHE II and SAPS 3 scores toward increased value and in-hospital mortality after adjustment (relative risk - RR of 1.11 [1.07 - 1.14]; p < 0.001 - AUC with 95%CI 0.779 (0.751 - 0.806); RR of 1.01 (1.00 - 1.01); p < 0.001; AUC with 95%CI 0.819 (0.795 - 0.843), respectively).

**Figure 1:**
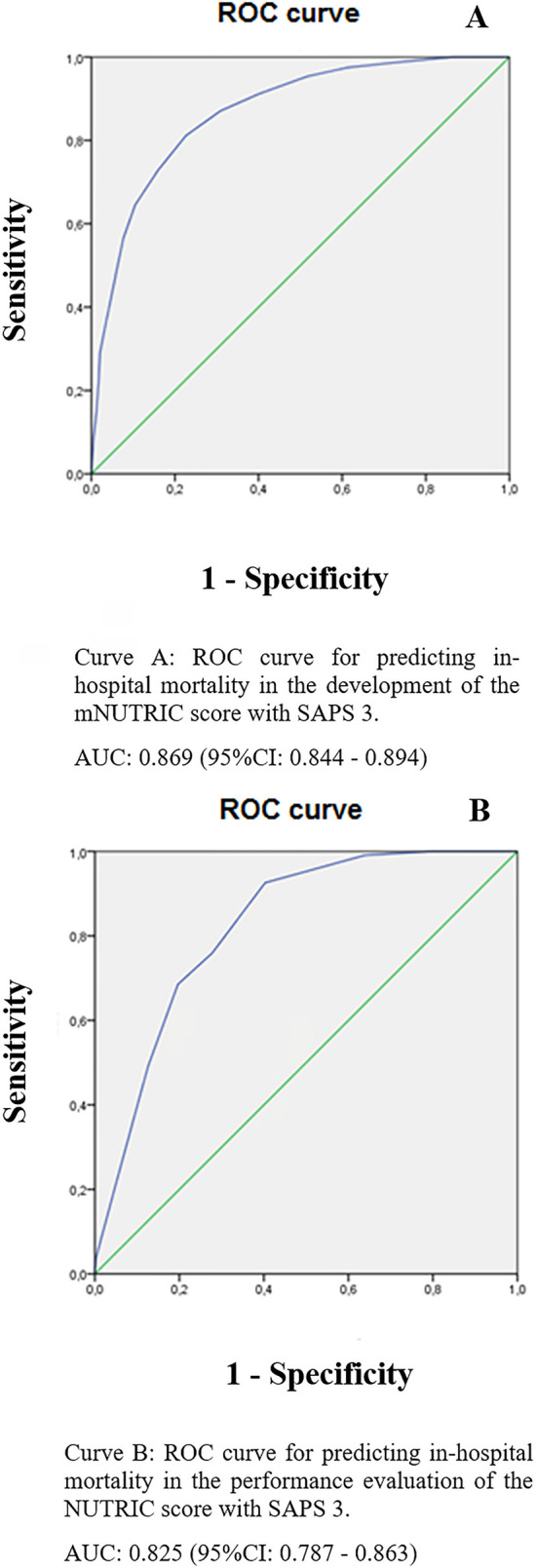
Receiver Operating Characteristic Curve for predicting in-hospital mortality in the development and performance evaluation of the modified NUTrition RIsk in the Critically ill score with Simplified Acute Physiology Score 3. ROC - Receiver Operating Characteristic; mNUTRIC - modified NUTrition RIsk in the Critically ill; SAPS 3 - Simplified Acute Physiology Score 3; AUC - area under the curve; 95%CI - 95% confidence interval.

Table 2 shows the mNUTRIC with SAPS 3. For development, data on the performance of the new instrument versus the mNUTRIC score in the study sample (n = 1,025) were as follows: correlation between scores of r = 0.839 (p < 0.001); agreement on nutritional risk classification between the instruments of k = 0.543 (p < 0.001); and the ability to predict in-hospital mortality from AUC resulted in an area of 0.869 (95%CI 0.844 - 0.894) (Figure 1). Data on the discriminative ability to predict 28-day mortality of the mNUTRIC score are described in table 2.

**Table 1:** Patients’ characteristics mNUTRIC - modified NUTrition RIsk in the Critically ill; SAPS 3 - Simplified Acute Physiology Score 3; APACHE II - Acute Physiology and Chronic Health Evaluation II; SOFA - Sequential Organ Failure Assessment; ICU - intensive care unit; AUC - area under the curve. *A 95% confidence interval was adopted for kappa agreement and Pearson correlation.

Characteristics	Model development (n = 1,025)	Model performance evaluation (n = 490)
Age (years)	72 (57 - 83)	72 (57 - 83)
Sex (n/%)		
Female	488 (47.6)	241 (49.2)
Male	537 (52.4)	249 (50.8)
APACHE II score	15 (11 - 20)	14 (11 - 18)
SOFA score	2 (1 - 5)	2 (1 - 5)
SAPS 3 score	47 (37 - 59)	45 (35 - 56.2)
BMI (kg/m^2^)	25.2 (22 - 28.4)	25.1 (22.1 - 28.5)
Place of origin (n/%)		
Emergency department	440 (42.9)	187 (38.2)
Ward	135 (13.2)	65 (13.3)
Hemodynamic unit	41 (4)	19 (3.9)
Surgical unit	331 (32.3)	180 (36.7)
Semi-intensive care unit	39 (3.8)	24 (4.9)
Other	14 (1.4)	6 (1.2)
Transferred from another health care facility	24 (2.3)	9 (1.8)
Reason for ICU admission (n/%)		
Clinical condition	713 (69.6)	320 (65.3)
Surgery	296 (28.9)	156 (31.8)
Trauma	14 (1.4)	11 (2.2)
Burn	1 (0.1)	2 (0.4)
Unspecified diagnosis	0 (0)	1 (0.2)
ICU outcome (n/%)		
Discharge	907 (88.5)	438 (89.4)
Death	118 (11.5)	52 (10.6)
Hospital outcome (n/%)		
Discharge	778 (75.9)	380 (77.6)
Death	239 (23.3)	108 (22.0)
Length of hospital stay (days)	15 (7 - 32)	16 (7 - 30.2)
Length of ICU stay (days)	4 (2 - 8)	4 (3 - 7)
Use of MV (n/%)		
Yes	327 (31.9)	150 (30.6)
No	698 (68.1)	340 (69.4)

APACHE II - Acute Physiology and Chronic Health Evaluation II; SOFA - Sequential Organ Failure Assessment; SAPS 3 - Simplified Acute Physiology 3; BMI - body mass index; ICU - intensive care unit; MV - mechanical ventilation.

**Table 2:** Proposed modified NUTrition RIsk in the Critically ill score with Simplified Acute Physiology Score 3

mNUTRIC score variables	Proposed model with SAPS 3 score			mNUTRIC score	
Age (years)	**Interval**	**Score**		**Frequency**	**Score**
	< 50	0		< 50	0
	50 to < 75	1		50 to < 75	1
	≥ 75	2		≥ 75	2
SAPS 3 score	< 45	0	APACHE II score	< 15	0
	46 - 50	1		15 to < 20	1
	51 - 54	2		20 - 28	2
	> 54	3		≥ 28	3
SOFA score	< 6	0		< 6	0
	6 to < 10	1		6 to < 10	1
	≥ 10	2		≥ 10	2
Comorbidities	0 - 1	0		0 - 1	0
	≥ 2	1		≥ 2	1
Length of hospital stay before ICU (days)	0 to < 1	0		0 to < 1	0
	≥ 1	1			
Kappa agreement*	0.543 (< 0.001)				
Pearson correlation*	0.839 (< 0.001)				
AUC	0.869 (0.844 - 0.894)			0.783	
	**Performance evaluation (n = 490)**				
Kappa agreement	0.563 (< 0.001)				
Pearson correlation	0.804 (< 0.001)				
AUC	0.825 (0.787 - 0.863)				

mNUTRIC - modified NUTrition RIsk in the Critically ill; SAPS 3 - Simplified Acute Physiology Score 3; APACHE II - Acute Physiology and Chronic Health Evaluation II; SOFA - Sequential Organ Failure Assessment; ICU - intensive care unit; AUC - area under the curve. *A 95% confidence interval was adopted for kappa agreement and Pearson correlation.

The performance of the proposed model was evaluated using one-third of the sample (n = 490). The agreement between the instruments (mNUTRIC composed of SAPS 3 *versus* mNUTRIC score) was 0.563 (p < 0.001); the correlation was 0.804 (p < 0.001); and the discriminative ability of the proposed model to predict in-hospital mortality was AUC of 0.825 (95%CI 0.787-0.863) (Figure 1).

Patients classified as high nutritional risk in the proposed model showed an incidence ratio (IR) for in-hospital mortality of 1.263 (95%CI 1.178 - 1.353; p < 0.001) in the analysis after adjusting for age and sex. Similarly, the predictive validity of the mNUTRIC score showed a higher IR for in-hospital mortality in patients with high nutritional risk (IR 1.321; 95%CI 1.231 - 1.417; p < 0.001).

## DISCUSSION

In this study, we hypothesized that APACHE II substitution by SAPS 3 in the mNUTRIC score would result in a comparable accuracy for all-cause in-hospital mortality prediction. Our data show good performance with regard to the ability to predict in-hospital mortality after adjusting for age and sex, as well as discriminative ability for in-hospital mortality. These results strongly relate to the results of both the original NUTRIC study (AUC: 0.783)^(1)^ and its modified version (AUC: 0.768) for mortality.^(2)^

The NUTRIC scoring system is the first specific tool for ICU nutritional screening and can be easily applied to critically ill patients as long as other variables, such as the APACHE II and SOFA scores, are available when patients are admitted to an ICU.^(10)^ It was created for nutritional screening, but it has proven to be an effective predictor of mortality in patients at nutritional risk.^(11, 12)^ Currently, prognostic scores that are more suitable for ICU settings have been used, such as the SAPS 3.^(13, 14)^ Therefore, knowing whether the SAPS 3 could replace the APACHE II in the NUTRIC system without compromising performance would provide a quick option for screening this specific group of patients. To the best of our knowledge, this study was the first to evaluate the validity of the replacement of the APACHE II with the SAPS 3 as a severity marker in the mNUTRIC.

The model was developed using robust statistical modeling and sampling. However, this study has several limitations. In Heyland’s original NUTRIC study,^(1)^ the median patient age was 63.5 years, while in our study, the median patient age was 72 years. The APACHE II was 21, while in our sample, it was 15. Likewise, the SOFA in Heyland’s study was 7, while in our study, it was 2, indicating that our patients, despite being older, had lower disease severity. Although the severity scores were lower in our sample, the mean length of ICU stay was four days, which indicates nutritional risk and requires the initiation of nutritional therapy.^(15)^ Our study was conducted retrospectively in a single center, although the NUTRIC score was obtained prospectively on ICU admission; therefore, it is still necessary to apply it in other ICUs with patients with more severe illness and to perform a prospective performance evaluation. Undoubtedly, future studies are needed for external performance evaluation of the proposed model. We believe the major limitation of this study was the absence of nutritional adequacy data analysis. The nutritional data unavailability precluded evaluation of the model proposed and its response to nutrition support. This limitation is relevant because the NUTRIC score was designed to evaluate which patients benefit most from nutritional therapy; thus, this analysis is crucial for performance evaluation of the model. Nevertheless, we believe that this study contributed to providing initial evidence on the possibility of substitution of APACHE II in the mNUTRIC score by SAPS 3 as a marker of severity without impairing its performance in predicting mortality.

## CONCLUSION

Our data suggest that the substitution of APACHE II by SAPS 3 as a severity marker in the mNUTRIC score showed good performance in predicting in-hospital mortality. These data provide the first evidence regarding the validity of the substitution of APACHE II by SAPS 3 in the mNUTRIC as a marker of severity. Multicentric studies and additional analysis of nutritional adequacy parameters are required.
